# Lead Exposure Assessment and Its Impact on the Structural Organization and Morphological Peculiarities of Rat Ovaries

**DOI:** 10.3390/toxics11090769

**Published:** 2023-09-10

**Authors:** Adam Osowski, Larysa Fedoniuk, Yaroslav Bilyk, Olena Fedchyshyn, Mykhailo Sas, Solomiia Kramar, Yuliia Lomakina, Volodymyr Fik, Sofija Chorniy, Joanna Wojtkiewicz

**Affiliations:** 1 Department of Pathophysiology, University of Warmia and Mazury in Olsztyn, 2 Oczapowskiego Street, 10-719 Olsztyn, Poland; 2 Histology and Embryology Department, I. Horbachevsky Ternopil National Medical University, 1 Maidan Voli Street, 46001 Ternopil, Ukraine; 3 Department of Medical Biology and Genetics, Bukovinian State Medical University, 15 Yu. Fedkvich Street, 58000 Chernivtsi, Ukraine; 4 Department of Normal Anatomy, Danylo Halytsky Lviv National Medical University, 69 Pekarska Street, 79010 Lviv, Ukraine

**Keywords:** rat ovaries, lead toxicity, morphology of ovaries, light microscopy

## Abstract

Lead is known to be highly toxic to humans, causing various disorders infetal development. An experiment was conducted to examine the effects of lead acetate on the structural organization of female rat ovaries. The study involved 40 non-linear female rats divided into four groups: a control group, a low-dose group, a moderate-dose group, and a high-dose group. The rats were given lead acetate solutions in varying doses for 30 days, and their ovarian tissue was examined using light microscopy.The results showed that increasing doses of lead acetate led to morphological changes in the cortex and medulla of the rat ovaries. The changes were characterized by a decrease in ovarian mass, alterations in the thickness of the tunica albuginea (protein envelope), and a reduction in the number of follicles. Light microscopy revealed that exposure to lead acetate resulted in a significant decrease in the number of follicles in all experimental groups, with the high-dose group experiencing the most significant decrease.These findings suggest that lead acetate has a dose-dependent negative impact on the morphology and function of female rat ovaries. Further studies are needed to investigate the potential impact of lead on human ovarian tissue.

## 1. Introduction

The harmfulness of lead to humans is determined by its significant toxicity and high cumulative capacity [1,2,3,4]. There are almost no functions in the human body that are not exposed to the toxic effects of lead [5,6,7,8,9,10].Lead has prominent membrane-toxic properties, it can change the activity of enzymes, influence biochemical processes, and is capable of accumulation; with long-term exposure, lead causes long-term negative bioeffects [2,11,12,13]. Lead belongs to the poisons with a polytropic mechanism of action, which is manifested by specific toxic effects on hematopoietic organs, lesions of the central and peripheral nervous system, and effects on the gastrointestinal tractand the cardiovascular and immune systems. It harms the liver and kidneys, disrupts metabolic processes, including protein synthesis, and also has embryotoxic effects [14,15,16,17].

The female reproductive system is greatly affected by exposure to environmental toxicants [18]. Lead, being one such reproductive toxicant, can affect the gonadal structure and functions and can cause alterations in fertility [19]. Reproduction, a vital process for species continuity, is significantly impacted by widespread pollutant use, including lead acetate. In females, toxin exposure disrupts ovarian physiology, affecting reproduction. The ovary, a complex mix of germs and somatic cells, regulates follicle formation, oocyte development, ovulation, and corpus luteum formation. Lead’s influence can disrupt these processes, causing pathological changes [20].It disrupts hormone synthesis, follicular maturation, ovulation, and ovarian cycle, resulting in reduced fertility, prolonged conception time, spontaneous abortion, stillbirths, and developmental defects. Pollutant-induced ovarian toxicity is caused by endocrine disruption and oxidative stress. Oxidative stress arises from suppressed antioxidant defenses, triggering reactive oxygen and nitrogen species, which cause DNA damage and activate apoptotic and inflammatory markers [21].

He Y. et al. detected ovarian histopathological damage in rats exposed to lead [22]. Dumitrescu et al. showed that lead also disturbed the release of hormones related to animal reproduction, such as through the decrease in follicle-stimulating hormones, estradiol, progesterone and the increase in luteinizing hormones and testosterone in female rats [23]. Moreover, lead disrupted spermatogenesis and steroidogenesis in mammal reproductive organs [24]. The effects on the physiology, histomorphology, development, and biomarkers have been observed on different organs of animals and humans. There is almost no function in the human body that is not affected by lead toxicity [25].

Lead as an environmental pollutant and classic toxic agent remains at the center of the attention of ecologists, toxicologists, hygienists, morphologists, and clinicians from different areas of medicine and biology [5,6,7,9,10]. This is evidenced by the fact that for within comparatively short period, the amount of this potentially toxic chemical substance increased dozens, and even hundreds, of times in the environment and has become a global issue [1,2,3,4,26].It is known that rats treated with 0.1% w/v lead acetate exhibit demyelination, collagenous scar formation, and neuronal atrophy in the hippocampal region. This treatment induces oxidative stress, which plays a crucial role in the brain damage of observed animals [27].

The main aim of the work was to study the effect of low, middle, and high doses of lead acetate on the structural organization of the ovaries of female rats in the experiment.The result of exposure to lead affecting the reproductive system is of particular interest, as it leads to various disorders of fetal development, which is confirmed by experimental and clinical studies [18,19,20,21,22,23,24,25,26,27].

## 2. Materials and Methods

The research was carried out at the Central Scientific-Research Laboratory at I. HorbachevskyTernopil National Medical University, the Ministry of Health of Ukraine (attestation certificate № 053/13 dated 4 March 2013 to 3 March 2018, technical competence certificate №001/18 dated 26 September 2018 to 28 December 2023).

All experiments were conducted in the first half of the day on special premises at a temperature of 18–22 °C, relative humidity of 40–60%, and light of 250 lux. The experiments were carried out in keeping with the requirements of the European Convention for the Protection of Vertebrate Animals used for Experimental and other Scientific Purposes (Strasbourg, 18 March 1986), the Resolution of the First National Congress on Bioethics (Kyiv, 2001), and the Order of the Ministry of Health of Ukraine № 690 dated 23 September 2009, the Law of Ukraine “On Protection of Animals against Cruel Treatment” (2006). The Bioethics Committee of I. HorbachevskyTernopil National Medical University, the Ministry of Health of Ukraine (minutes № 1 dated 4 January 2021), did not find any violations of ethical norms during the conductof the study.

The study involved 40 non-linear female rats, aged 4 months, and weighing 180–210 g. They were divided into four experimental groups (Table 1): Group I (control), Group II (0.5 mg/kg), Group III (10 mg/kg), and Group IV (60 mg/kg). All groups of animals received lead acetate solution for drinking for 30 days. The rats were kept under standard vivarium conditions with free access to food and water (water was given after administering the lead acetate solution).We considered establishing a dose–response relationship of lead acetate on the toxicity in rat ovaries. This involves using a range of doses to determine the range at which adverse effects become significant or to observe a clear trend in the response. Doses that span a significant scope of concentrations can help establish the dose–response relationship. The results in our manuscript showed that increasing doses of lead acetate from 0.5 mg/kg to 60 mg/kg led to morphological changes in the cortex and medulla of the rat ovaries [27,28,29].

The rats were kept under standard vivarium conditions with free access to food and water (water was given after an animal had taken lead acetate solution).

Euthanasia of rats was performed using total bloodletting from the heart after preliminary sodium thiopental narcosis (60 mg kg^−1^ of the bodyweight into the peritoneum). After the animals were removed from the experiment, their blood was taken for biochemical examination and their ovaries for histological examination. The material was taken at the same time of the day from 11 a.m. to 3 p.m., on the premises with a temperature of 18–20 °C.

Excised ovaries were processed for the light microscopic observation, according to the standard procedures. After the ovary was removed, it was weighed and small pieces were cut from the middle part of the organ. The material was fixed for 24 h in a phosphate-buffered 10% formalin solution, after which pieces were embedded in paraffin wax. Paraffine-embedded microtome sections 5 μm thick were stained with hematoxylin&eosin. The specimens were examined under the light microscope «Nikon Eclipse Ci» (Tokyo, Japan), using the lenses ×4, 10, 20, and the eyepiece ×10. The pictures of the histological specimens were taken by the Sigeta digital camera (Hangzhou, China).

The biochemical research methods also included the determination of diene conjugate concentration (DC), concentration of TBA-active products, and the indicators of endogenous intoxication.

The concentration of DC was determined through the method based on the fact that hydroperoxides extracted with heptane-isopropyl mixture have a certain maximum absorption: DC at λ = 232 nm. The optical density was determined on a SF-46 LOMO spectrophotometer (St. Petersburg, Russia)

The control was a sample that had 0.2 mL of distilled water instead of the test serum. The calculation of the content of DC was performed according to the formula C = E · V1/V2, where E—the optical density of the heptane layer of the sample, V 1—the end volume of heptane extract (4 mL), V2—the volume of research material (2 mL). The content of diene conjugates was expressed in units per liter.

The principle of the method is as follows: in an acidic environment at high temperatures, the substances react with thiobarbituric acid, forming a colored complex with a maximum absorption at a wavelength of 535 nm.

Initially, 1 mL of serum was poured into centrifuge tubes and 2 mL of 30% trichloroacetic acid solution, and 0.1 mL of 5 M HCl and 2 mL of thiobarbituric acid were added and placed in a boiling water bath for 15 min. Then, they were cooled and centrifuged at 3000 rpm for 10 min. The supernatant was photometered on a SF-46 spectrophotometer at 535 nm.

The content of TBA-active products was calculated based on the molar extinction coefficient of the colored complex, which is equal to 1.56 × 105 cm^−1^ M^−1^ and expressed in micromoles per liter (μmol/L).

Endogenous intoxication was assessed by the determination of molecules of middle weight (MMW). The content of the MMW was determined by a method based on precipitation of serum proteins with 10% trichloroacetic acid, and the optical density of the supernatant was measured on a spectrophotometer SF-26 at a wavelengths of 280 nm, 260, 254, and 238 nm (MMW280, MMW260, MMW254, MMW238). Concentrations were obtained based on extinction measurements.

## 3. Results

**Figure 1:**
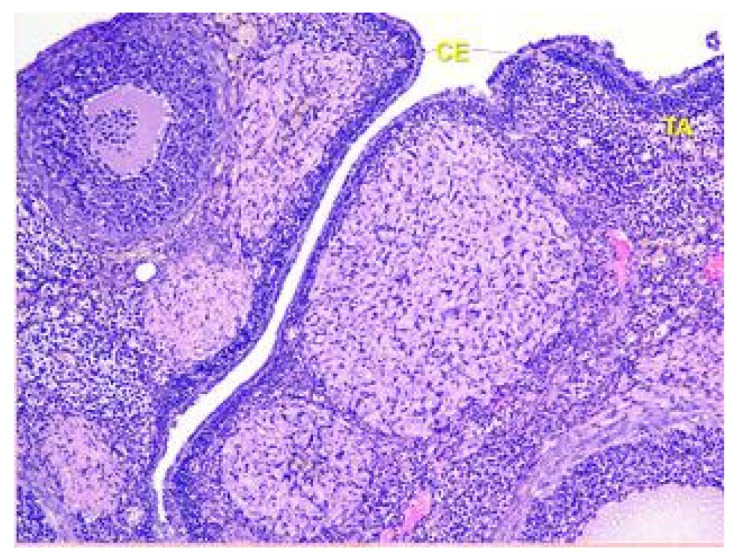
Structural organization of the ovary of an animal of the control group. Fibrous tunica albuginea (TA) and a single layer of the cuboidal epithelial (CE) cells on the surface of the ovary. Stained with Hematoxylin and Eosin. ×40.

The surface of the ovaries in animals in the control group is covered mainly by a single layer of cuboidal epithelium. But also visible are placesthat are covered by squamous epithelium. On the apical surface of epithelial cells, microvilli are present (see Figure 1).

**Figure 2:**
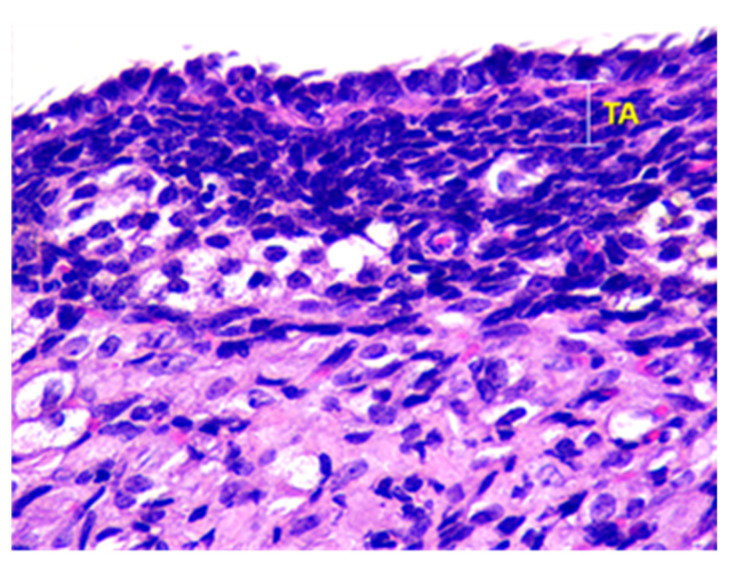
Structural organization of the ovary of an animal of Group I. Fibrous connective tissue layer—tunica albuginea (TA). Stained with Hematoxylin and Eosin. ×100.

Beneath the epithelium of the ovary, there is a thin capsule called the tunica albuginea, which is mainly composed of collagen and elastin fibers, along with a small number of myocytes (see Figure 2).

The ovary consists of an external cortex and an internal medulla, each having their own unique structures.

In rats of Group I, the cortex of the ovaries is composed of a connective tissue stroma that is highly cellular and where the follicles are embedded. The stroma contains numerous collagen fibers and a small number of elastic fibers.

**Figure 3:**
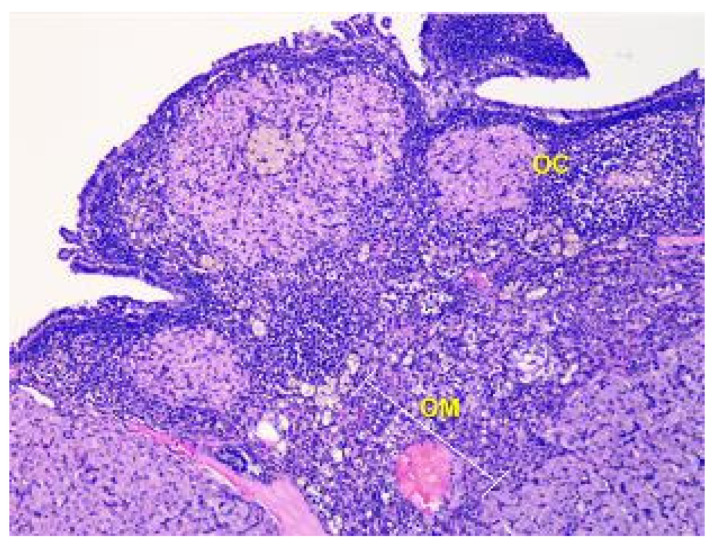
Structural organization of the ovary of an animal of the control group. The peripheral zone of the stroma-cortex (OC) and central zone of the ovarian stroma–medulla (OM). Numerous follicles of various sizes and stages of their development in the cortex. Stained with Hematoxylin and Eosin. ×100.

The parenchyma of the ovary contains different types of follicles, including primordial, primary, secondary, and tertiary follicles, as well as atretic follicles and a few corpus luteum. In addition, a significant number of blood vessels are present (see Figure 3).

Primordial follicles are found in the cortex just beneath the tunica albuginea. Primordial follicles are limited to a thinning peripheral rim. The oocyte is surrounded by a single layer of flattened follicular cells, and their nuclei are positioned eccentrically and appears light with a prominent nucleolus.

In the primary follicle, the oocyte is surrounded by a cuboidal epithelium with granular cytoplasm. The zona pellucida becomes visible, and the parenchymal cells of the ovary surrounding the growing follicle become organized in concentric sheaths called theca folliculi.

The secondary follicle appears as small fluid-filled spaces between the granulosa cells, which enlarge and fuse to form the follicular antrum, the defining feature of the secondary follicle. The immature secondary follicles are characterized by having plump granulosa cells, plump thecal cells, and little discernment between these two layers. The oocyte is located eccentrically in the follicle, surrounded by granulosa cells in the cumulus oophorus. With the continued growth of the follicle, the theca folliculi differentiates into a theca interna and theca externa. Other features of the immature follicles are poor adhesion between granulosa cells and the primary oocyte and the absence of a zona pellucida. The theca externa retains the characteristics of highly cellular connective tissue with smooth muscle cells.

**Figure 4:**
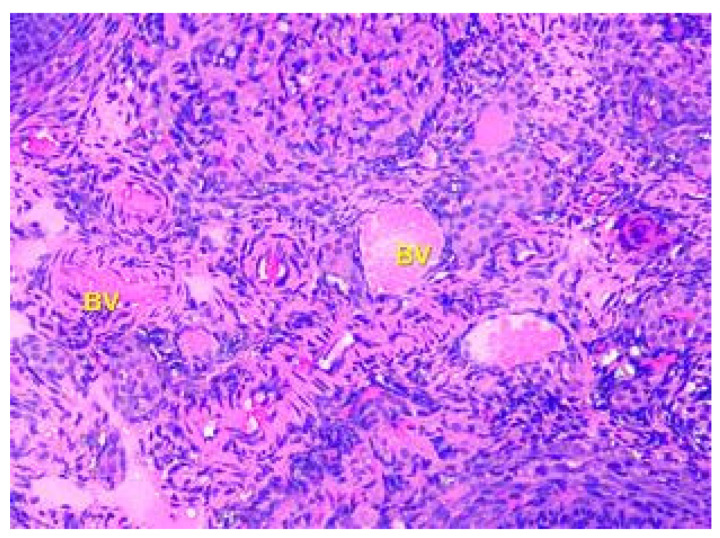
Structural organization of the ovary of an animal of the Group I control group. The ovary medulla, which contains blood vessels (BV), elastic fibers, and nerve endings. Stained with Hematoxylin and Eosin. ×100.

The tertiary or preovulatory or Graafian follicle forms a small “bump” on the surface of the ovary (*Macula pellucida*). It is characterized by a thinning of the capsule. The oocyte is floating freely in the follicular antrum. It is still surrounded by granulosa cells which form the corona radiata. The follicle finally ruptures at the stigma and the oocyte is released from the ovary. The medulla is composed of loose connective tissue, which contains blood vessels and nerves, which are displayed in Figure 4.

**Figure 5:**
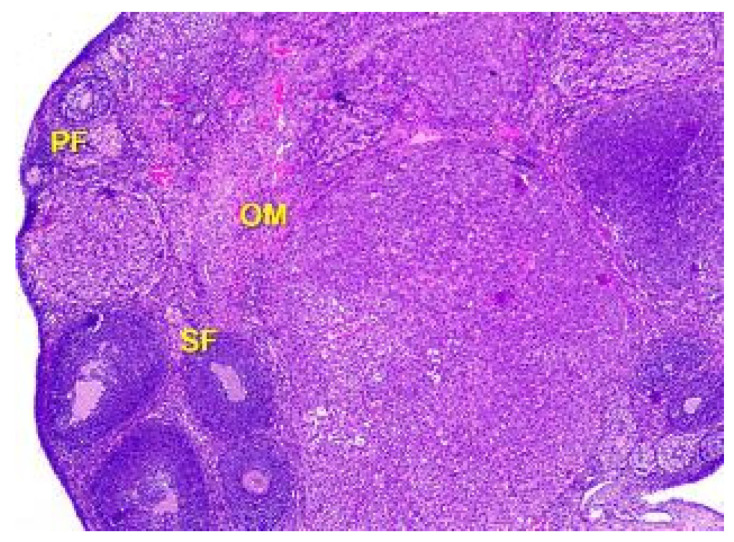
Structural organization of the ovary cortex in an animal under the influence of a dose of 0.5 mg/kg of the body weight. Follicles in the cortex at different stages of the development: primary follicles (PF), secondary follicles (SF), Graafian follicles (GF). Blood vessels, elastic and collagen fibers in connective tissue stroma of ovarian medulla (OM). Stained with Hematoxylin and Eosin. ×40.

Upon histological examination of the ovaries of rats in Group II, it was found that the structural organization of the ovaries in the experimental animals showed no significant differences from those in the control group, as depicted in Figure 5. The cortex of the ovary contained primordial, primary, secondary, and tertiary follicles. The primordial follicle contained a primary oocyte, which was surrounded by a single layer of flattened pregranulosa cells. In the primary follicle, the primary oocyte was surrounded by a single layer of cuboidal granulosa cells. The secondary follicle was characterized by a well-defined transparent shell, with the primary oocyte surrounded by more than one layer of granulosa cells. In the tertiary follicle, the primary oocyte was surrounded by more than one layer of granulosa cells and had a fluid-filled space. A mature (preovulatory) follicle had a fully formed cavity with follicular fluid, in which the egg floated freely.

**Figure 6:**
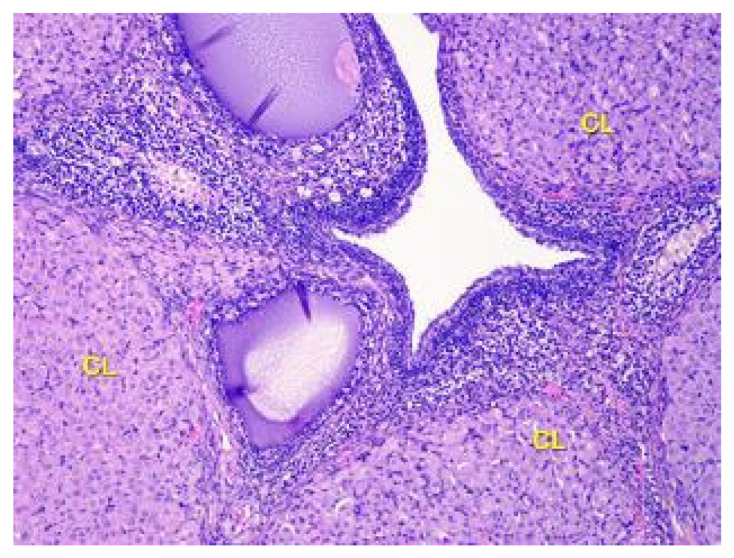
Structural organization of the ovary cortex in ananimals of Group II. Active and degenerating corpora lutea (CL) in the cortex. Stained with Hematoxylin and Eosin. ×40.

It was discovered that there is a higher number of corpus luteum in the ovarian cortex of the second group of rats compared to the control animals. While the newly formed corpus luteum from the previous ovulation reaches its maximum size, degenerate corpora lutea are also present. Vacuoles are commonly observed, especially in the cells at the center of the large corpus luteum, indicating active steroidogenesis (as shown in Figure 6). The newly formed corpus luteum is small, with cells exhibiting a basophilic cytoplasm and sometimes a central fluid-filled cavity retained from the follicular stage.

**Figure 7:**
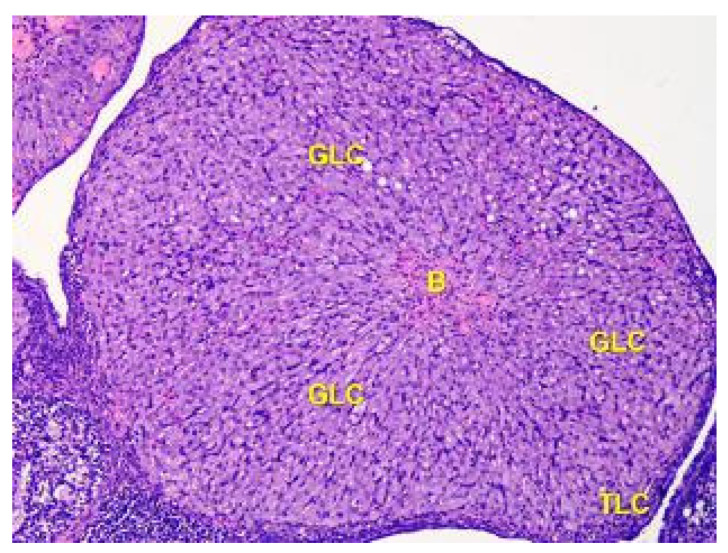
The structure of the corpus luteum in the cortex of the ovary of the Group II animals. The remnant blood clot (B) in the center of the corpus luteum is surrounded by a broad zone of granulosa luteal cells (GLC), and a thin zone of theca luteal cells (TLC). Stained with Hematoxylin and Eosin. ×100.

The corpus luteum is made up of luteocytes, which have an irregular shape, with nuclei that are stained basophilic in the center of the cell and eosinophilic cytoplasm. In Figure 7, it can be observed that the size of luteocytes varies depending on their location within the corpus luteum. The periphery is dominated by small, star-shaped cells with large oval nuclei.

**Figure 8:**
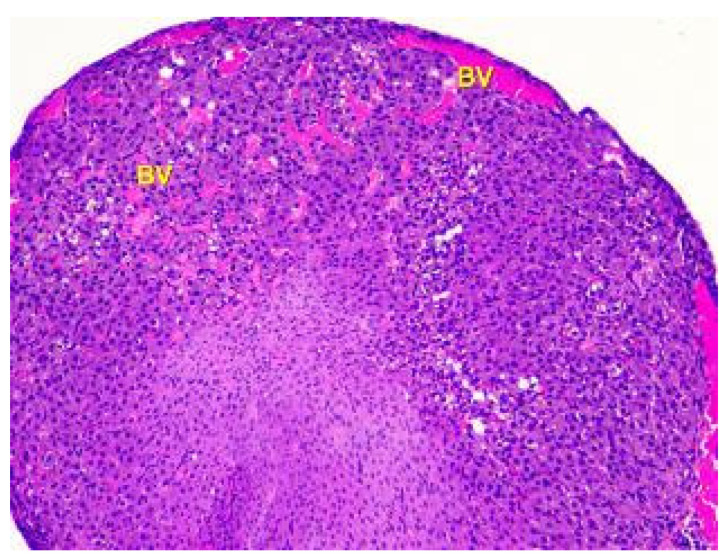
Histological structure of the ovary of an animalunder the influence of lead acetate in dose of 10.0 mg/kg of the body weight. Blood-filled vessels (BV) in the cortex and medulla. Stained with Hematoxylin and Eosin. ×100.

Histological examination of the ovaries of animals in the third group showed that the structural components of the cortex and medulla have changed, particularly the blood vessels. Ovaries of animals given low doses of lead acetate exhibited moderately increased blood supply to the cortex and medulla. Light microscopy revealed an uneven blood supply of vessels with perivascular edema, as shown in Figure 8. This indicates an increase in vascular permeability.

**Figure 9:**
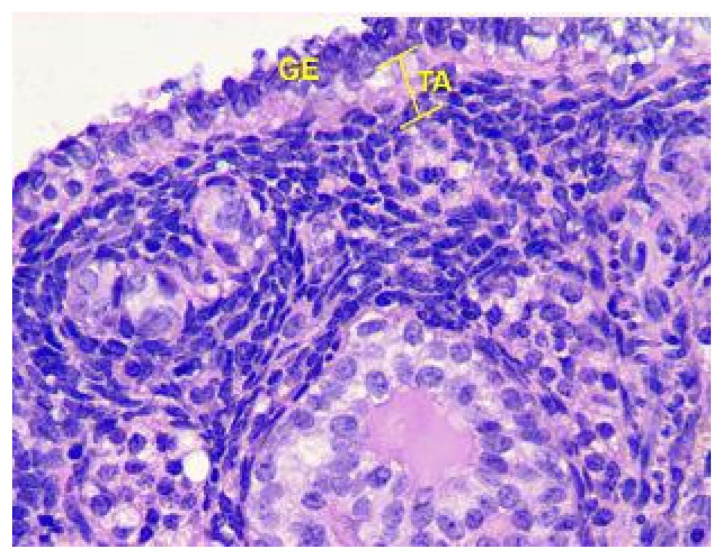
Structural organization of the ovarian cortex ofGroup III animals. *Fibrous tunica albuginea* (TA) and the layer of germinal epithelium (GE). Stained with Hematoxylin and Eosin. ×400.

Analysis of the follicles in the cortex of the ovaries, as shown in Figure 9, revealed that in Group III animals, there was a slight increase in the number of primordial follicles in non-ovulating ovaries, an increase in the thickness of the *Tunica albuginea*, and a slight decrease in atretic follicles compared to the control.

The initiation of follicle growth is an irreversible process that results in gradual reproductive aging. However, the gradual activation of primordial follicles provides a balance between the constant recruitment of follicles before the estrous cycle and the simultaneous limitation of premature depletion of the ovarian reserve.

**Figure 10:**
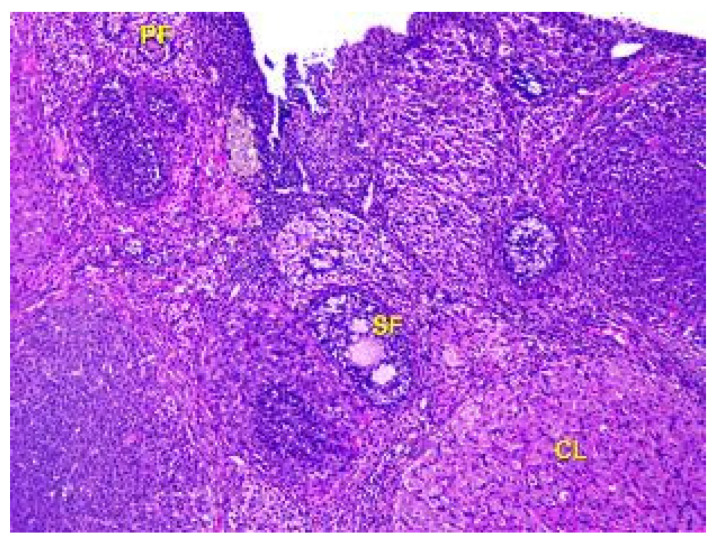
Structural organization of the animal’s ovary under the influence of lead acetate in dose of 60 mg/kg of the body weight (Group IV model group). Primary follicles (PF), secondary follicles (SF), and corpus luteum (CL) in the cortex of the organ. Stained with Hematoxylin and Eosin. ×100.

Upon microscopic examination of the ovaries of rats in Group IV, it was found that the number of follicles at all stages of growth and development in the cortex had decreased. Only a few primary and secondary follicles were visible, and there was a small number of primordial follicles located at the periphery of the ovarian cortex, along with a lower number of tertiary follicles. The corpus luteum was located chaotically in the parenchyma of the organ, as shown in Figure 10.

The results of the microscopic examination revealed that the ovaries of rats exposed to lead acetate showed significant morphological changes in both the cortex and medulla. In the cortex, there was a decrease in the number of follicles at all stages of growth and development, including a reduced number of primordial and tertiary follicles. Furthermore, the ovaries of rats exposed to low doses of lead acetate showed a moderately increased blood supply of the cortex and medulla, which was accompanied by an uneven blood supply to vessels with perivascular edema, suggesting an increase in vascular permeability.

The analysis of lipid peroxide oxidation (Table 2) in animals of Group II revealed a 4.7 times increase in the DC level (*p* < 0.001) compared with animals in the control group, and 2.9 times in TBA-active products (*p* < 0.001), respectively.

Animals inGroup IV, which were given large doses of lead, causing intoxication in the body, showed an increase in DC of 6.3 times (*p* < 0.001), compared with animals in the control group, and 3.7 times for TBA-active products (*p* < 0.001), respectively.

When comparing the degree of toxic effects of lead on the processes of lipid peroxidation, it was found that in Group IVanimals compared with Group IIIanimals, DCs were 55.8% higher (*p* < 0.001), and TBA-active products were 24.8% higher (*p* < 0.001).

Thus, lead acetate causes dose-dependent activation of lipid peroxidation processes in rats.

Since the experiments showed an increase in oxidation processes, it was advisable to study the content of MMW (Table 3).

In the Group IIIanimals, compared with the control, there was an increase in all types of MMW: MMW238 by 36.4% (*p* < 0.001), MMW254 by 85.8% (*p* < 0.001), MMW260 by 77.5% (*p* < 0.001), and MMW280 increased 2.2 times (*p* < 0.001).

In the Group IVanimals, compared with the control, there was also an increase in all types of MMW: MMW238 increased by 67.1% (*p* < 0.001), while MMW254 increased 2.2 times (*p* < 0.001), MMW260 2.1 times (*p* < 0.001), and MMW280 2.6 times (*p* < 0.001).

It was found that all MMW types increased with increasing doses of lead acetate. Comparing Groups III and IV, in the latter higher indicators were found: MMW238 by 22.5% (*p* < 0.01), MMW254 by 17.2% (*p* < 0.05), MMW260 by 16.1% (*p* < 0.05), and MMW280 by 12.8% (*p* < 0.05).

Therefore, only large doses of lead acetate cause an increase in MSM.

Overall, these findings suggest that lead acetate exposure may have harmful effects on ovarian function and fertility in rats by damaging the growth, maturation, and development of follicles. The changes in ovarian surface structures, along with changes in blood supply and vascular permeability, may contribute to this damage. These results underscore the need for further research into the long-term consequences of lead acetate exposure on reproductive health in both animals and humans.

## 4. Discussion

Lead remains a significant environmental concern, as it is a hazardous chemical element that contaminates and harms the human body. Classified as a potent poison, lead can have far-reaching effects, leading to toxicity in blood-producing organs and damage to the central and peripheral nervous systems, digestive tract, cardiovascular system, and immune system. Furthermore, it negatively affects the liver and kidneys, disrupts metabolic processes like protein synthesis, and poses risks to fetal development [2,8,30,31,32,33]. Its membrane-toxic properties, ability to modify enzyme activity, impact on biochemical processes, bodily accumulation, and persistence in causing harmful long-term effects are well-documented [2,11,13].

The reproductive system of both males and females is also affected by lead [13,15,31,34,35,36,37]. It specifically affects the condition of the ovaries in complexways. Notably, several primary molecular mechanisms have emerged as pivotal contributors to ovarian toxicity. The most common molecular mechanism is oxidative stress, when lead acetate exposure incites the generation of reactive oxygen species (ROS), disrupting the delicate balance between oxidants and antioxidants within ovarian tissues. This oxidative stress triggers cellular damage to critical components such as lipids, proteins, and DNA, culminating in compromised ovarian function and overall health [38]. Additionally, elevated rates of apoptosis, or programmed cell death, among ovarian cells are closely linked to lead acetate exposure. This phenomenon disrupts the natural progression of ovarian follicle development, potentially leading to a reduction in ovarian reserves and consequent fertility impairment [39]. Aside from that, lead acetate can interfere with hormonal balances in the body, including disrupting the hypothalamic–pituitary–gonadal axis. This disruption can lead to altered hormone levels, which may impact ovulation, menstrual cycles, and fertility [40]. Also, it is proven that lead acetate can cause direct damage to DNA, potentially leading to mutations and genomic instability in ovarian and other cells [41,42].

In males, sperm count is reduced and other changes occur in the volume of sperm when blood lead levels exceed 40 μg/dL. Activities like motility and the general morphology of sperm are also impacted at this level [16,30]. The detailed mechanism of how lead induces male infertility has been reviewed [15], including mitochondrial dysfunction with decreased energy production and impaired cellular processes caused by lead acetate exposure [43].The problems with the reproductivity of females due to lead exposure are more severe than in males. Lead exposure affects female reproduction by impairing menstruations, such as amenorrhea, dysmenorrhea, and menorrhagia, reducing fertility potential, delaying conception time, altering the hormonal production and circulation, and affecting pregnancy and its outcome [44,45]. There may also be a period of sterility, and if pregnancy does occur, the chances of miscarriage and stillbirth are very high. If living children are born, they are usually small, weak, and develop slowly [3,8,37,46]. A large proportion of them die in their first year, and the survivors may be mentally retarded. These observations have given rise to regulatory laws for the employment of women in occupations with lead hazards.

Lead exposure impacts the reproductive systems of both males and females [13,31,37]. In males, blood lead levels surpassing 40 μg/dL lead to reduced sperm count, changes in sperm volume, and compromised sperm motility and morphology [16,30]. Although the precise mechanism of lead-induced male infertility remains under investigation, previous studies have delved into this issue [15].

In comparison, the impact of lead exposure on female reproduction is even more profound. Lead disrupts menstrual cycles, causing issues like amenorrhea, dysmenorrhea, and menorrhagia, while also diminishing fertility potential, delaying conception, altering hormone production and circulation, and affecting pregnancy outcomes [32,33]. This exposure might lead to periods of sterility, substantially increasing the risks of miscarriage and stillbirth if pregnancy does occur. Infants born in such circumstances are often undersized, frail, and slow to develop [3,8,37,46]. A significant number of them do not survive their first year, and survivors may face developmental challenges.

In preclinical studies evaluating the safety of drugs and chemicals, many are found to interfere with reproductive function in female rats. Such interference can manifest as changes in the normal morphology of the reproductive tract or disruptions in the duration of specific phases of the estrous cycle. To recognize these alterations, pathologists must have knowledge of the continuously changing histological appearance of the various components of the reproductive tract during the cycle and must accurately and consistently ascribe individual tracts. Although comprehensive reports illustrating the normal appearance of the tract during the rat estrous cycle have been available for many years, these changes can be difficult to discern [32,37,47].

Several studies have demonstrated that lead exposure can adversely affect the reproductive system of both males and females [31,32,33,34,48,49]. In males, blood lead levels exceeding 40 μg/dL can cause a reduction in sperm count and changes in sperm volume, motility, and morphology [15]. Lead exposure can also have severe effects on female reproductive health, including an increased risk of miscarriage, prematurity, low birth weight, and developmental problems during childhood [19,50,51,52,53]. Maternal blood lead levels are typically similar to those of infants, as lead can pass from the mother’s blood to the fetus through the placenta and breast milk. Lead primarily targets testicular spermatogenesis and sperm in the epididymis, inducing reproductive toxicity in males [53]. Previous studies have reported suppressed rates of serum testosterone, intratesticular sperm counts, and reduced sperm production rates in lead-treated groups [30]. The detailed mechanisms by which lead induces male infertility have been reviewed [15]. Moreover, lead has been shown to accumulate in bones, and during pregnancy, metabolic changes can mobilize the lead from bones into the blood, which increases the risk of lead toxicity [54]. However, increased calcium intake during pregnancy can help mitigate this phenomenon [3].

Small doses of lead acetate (0.05 mg/kg) mainly cause embryotoxic effects [47,55], leading to increased mortality of embryos and fetuses, although no teratogenic effects have been identified [56]. Studies on the effects of large doses of lead were mainly conducted in the second half of the last century [15,30,46].

In female rats, animal lead acetate was used for 30 days, which is a short time, equating to six months in terms of human life expectancy. Exposure to large doses of lead acetate reduces antioxidant enzymes, which contributes to the activation of prooxidants and damages the body. The ovaries in rats decreased in size, which may be due to a decrease in hormonal activity [56]. They were hyperemic, indicating the development of inflammation. Signs of dystrophy were noted, including a decrease in the thickness of the superficial epithelium and tunica albugena (protein envelope). Such changes indicate the development of infertility. Microscopic examination showed that almost all follicles were absent, with single yellow bodies chaotically localized in the organ parenchyma. Biochemical changes were confirmed by morphological ones. Inflammation was also present, and the cytokine cascade was activated, raising the possibility of rapid tumor development, which requires further research.

The effects of small, medium, and large doses of lead acetate on morphological changes in the ovaries of rats were also studied. The development of oxidative stress, causing damage, was established. Changes induced by small doses were adaptive, while those induced by large doses were damaging [26].

Embryotoxic effects are mainly provoked by small doses of lead acetate (0.05 mg/kg) [47], which leads to increased mortality of embryos and fetuses, although no teratogenic effect has been identified [56]. The effect of large doses of lead on the body was mainly studied in the second half of the last century [15,30,46].

Given that the sexual cycle in female rats lasts 5 days, animal lead acetate was used for 30 days, which is quite a short term, since in terms of human life expectancy it is six months.

Exposure to large doses of lead acetate reduces antioxidant enzymes, which contributes to the activation of prooxidants and damages the body. In rats, the ovaries decreased in size, which may be due to a decrease in hormonal activity [56]. They were hyperemic, indicating the development of inflammation. Signs of dystrophy were noted, such as a decrease in the thickness of the superficial epithelium and tunica abuginea. Such changes indicate the development of infertility. Microscopic examination showed that almost all follicles were absent, and there were single yellow bodies that were chaotically localized in organ parenchyma. Therefore, biochemical changes are confirmed through morphological ones. Given the signs of inflammation in which the cytokine cascade is activated, the possibility of rapid tumor development requires more research.

Thus, the effect of small, medium, and large doses of lead acetate on morphological changes in the ovaries in female rats was studied. The development of oxidative stress, which causes damage, has been established. Under the influence of small doses, the changes are adaptive, and in the case of large doses, they are damaging.

Lead poisoning causes severe effects and is a matter of serious concern. It is preventable, and knowledge about the toxicity of lead and its harmful effects on the human body, along with insights into the biochemical and structural features of lead intoxication, remains a task for theoretical and practical medicine [15,30,46].

## 5. Conclusions

Lead acetate exposure in rats causes dose-dependent morphological changes in both the cortex and medulla of the ovaries, resulting in reduced ovarian mass, alterations in tunica albuginea thickness, and fewer follicles, ultimately affecting growth and maturation. Small doses of lead do not cause the development of endogenous intoxication, in contrast to high doses, which increase the formation of toxic compounds. These findings underscore the serious reproductive toxicity of lead, particularly in high doses, and emphasize the significance of preventative measures to diminish exposure to this hazardous substance.

## Figures and Tables

**Table 1:** Distribution of the experimental animals.

Group №	Characteristics of the Experimental Model Group	Number of Animals
I	Intact albino rats (control)	10
II	Lead acetate (0.5 mg/kg)	10
III	Lead acetate (10 mg/kg)	10
IV	Lead acetate (60 mg/kg)	10

**Table 2:** Changes in lipid peroxidation in animal serum under different doses of lead acetate (M ± m, n = 10).

Group	Experimental Conditions	Index	
		DC, Unit/mL	TBA-Active Products, μmol/L
I	Control	0.84 ± 0.03	2.50 ± 0.04
III	Lead acetate (10 mg/kg)	3.42 ± 0.16 *^,^**	7.34 ± 0.57 *^,^**
IV	Lead acetate (60 mg/kg)	5.33 ± 0.22 *^,^**	9.16 ± 0.43 *^,^**

Note: here and in the following Tables: *—indexes are reliable in comparison with the control group; **—indexes are reliable compared to Group III.

**Table 3:** Changes in the content of molecules of middle weight (units/L) in the serum of rats under the influence of lead acetate (M ± m, n = 12).

Group	Index			
	MMW238	MMW254	MMW260	MMW280
I Control	0.231 ± 0.003	0.254 ± 0.004	0.276 ± 0.004	0.194 ± 0.006
III Lead acetate (10 mg/kg)	0.315 ± 0.017 *^,^**	0.472 ± 0.021 *^,^**	0.490 ± 0.010 *^,^**	0.438 ± 0.011 *^,^**
IV Lead acetate (60 mg/kg)	0.386 ± 0.012 *^,^**	0.553 ± 0.013 *^,^**	0.569 ± 0.013 *^,^**	0.494 ± 0.003 *^,^**

Note: here and in the following Tables: *—indexes are reliable in comparison with the control group; **—indexes are reliable compared to Group III.

## Data Availability

Not applicable.
